# Freeze-Dried Donor Milk for Fortification of Mother’s Own Milk in Preterm Infants: A Preliminary Observational Study

**DOI:** 10.3390/nu17193057

**Published:** 2025-09-25

**Authors:** Niels Rochow, Gisela Adrienne Weiss, Katja Knab, Isabell Prothmann, Stefan Schäfer, Jasper L. Zimmermann, Anastasia Meis, Stefanie Lohmüller-Weiß, Kerstin Simon, Simone Schäfer, Julia Welsch, Christoph Fusch

**Affiliations:** 1 Department of Pediatrics, Paracelsus Medical University, Breslauer Str. 201, 90471 Nürnberg, Germany; niels.rochow@klinikum-nuernberg.de (N.R.); gisela.weiss@klinikum-nuernberg.de (G.A.W.); katja.knab@klinikum-nuernberg.de (K.K.); isabell.prothmann@klinikum-nuernberg.de (I.P.); stefan.schaefer@klinikum-nuernberg.de (S.S.); jasper.zimmermann@klinikum-nuernberg.de (J.L.Z.); anastasia.meis@klinikum-nuernberg.de (A.M.); stephanie.lohmueller-weiss@klinikum-nuernberg.de (S.L.-W.); kerstin.simon@klinikum-nuernberg.de (K.S.); simone.schaefer@klinikum-nuernberg.de (S.S.); julia.welsch@klinikum-nuernberg.de (J.W.); 2 Department of Pediatrics, Rostock University Medical Center, 18057 Rostock, Germany; 3 Department of Pediatrics, McMaster University, Hamilton, ON L8S 4L8, Canada

**Keywords:** freeze-dried human milk, lyophilized donor milk, preterm infants, human milk fortification, exclusive human milk feeding, neonatal nutrition, protein-to-energy ratio, anthropometric outcomes

## Abstract

**Background/Objectives:** Freeze-dried high-temperature short-time pasteurized human milk fortifiers offer potential for exclusive human milk feeding in preterm infants while providing necessary nutritional supplementation. However, clinical data on safety, tolerability, and growth outcomes remain limited. This study evaluated donor milk fortification compared to conventional bovine protein-based fortification. **Methods:** We conducted a prospective non-interventional observational cohort study with a retrospectively matched comparison cohort at University Children’s Hospital of Nuremberg. Preterm infants ≥ 30 weeks gestational age requiring mother’s own milk fortification were included. The exposed cohort (*n* = 32) received freeze-dried high-temperature short-time pasteurized donor milk fortifier at 1.6–4.8 g/100 mL of mother’s own milk; the matched comparison cohort (*n* = 32) received bovine protein-based fortifier. Primary outcomes included feeding tolerance, safety parameters, and anthropometric measurements. Cohorts were matched for birth weight (±10%), gestational age (±5 days), and fortified feeding. **Results:** Baseline characteristics were not significantly different: gestational ages 32.8 ± 1.0 versus 33.0 ± 1.2 weeks; birth weights 1900 ± 380 g versus 1840 ± 370 g. Excellent feeding tolerance was demonstrated across >3100 feedings. No necrotizing enterocolitis, abdominal complications, or serious adverse events occurred. Blood glucose, triglycerides, and urea remained normal. Birth weights, lengths, and head circumferences showed no significant differences. Discharge parameters including weight, length, head circumference, and length of stay were also not significantly different. **Conclusions:** Freeze-dried human milk fortification demonstrates excellent safety and tolerability in preterm infants ≥ 30 weeks gestational age, achieving anthropometric outcomes not significantly different to bovine protein-based fortification. However, the suboptimal protein-to-energy ratio may limit applicability for very low birth weight infants. Therefore, freeze-dried high-temperature short-time pasteurized human milk fortification is suggested to provide appropriate nutritional supplementation for preterm infants with a birth weight over 1500 g.

## 1. Introduction

Preterm birth (<37 weeks gestation) remains a major public health problem and is the leading cause of neonatal morbidity and mortality worldwide. In 2020, the global incidence was estimated at 9.9% [1]. These infants are at risk for postnatal growth restriction, largely due to cumulative nutritional deficits with feeding intolerance as a major contributing factor [2,3]. Poor postnatal growth is strongly associated with impaired neurodevelopment and an increased risk of long-term metabolic diseases such as obesity, diabetes, and hypertension [4]. Optimizing early nutritional management is therefore critical to support somatic growth and brain development during the stay at the neonatal intensive care unit.

Human milk represents the optimal nutritional source for newborn infants, providing essential macronutrients, micronutrients, and bioactive components that support healthy growth, development, and immune function [5,6,7]. The composition of human milk is uniquely adapted to meet the specific needs of human infants, with varying concentrations of proteins, lipids, carbohydrates, minerals, vitamins, and biologically active components that change dynamically throughout lactation [8]. Beyond its nutritional value, human milk contains numerous bioactive factors including immunoglobulins, lactoferrin, lysozyme, oligosaccharides, and growth factors that provide protection against infection and inflammation while supporting the development of the gut microbiome and immune system [9,10].

For preterm infants, the benefits of human milk feeding are particularly pronounced. Exclusive human milk feeding has been consistently associated with reduced rates of necrotizing enterocolitis, late-onset sepsis, bronchopulmonary dysplasia, and retinopathy of prematurity compared to formula feeding [11,12]. Additionally, human milk feeding is associated with improved neurodevelopmental outcomes, enhanced cognitive development, and reduced risk of metabolic disorders later in life [13]. These benefits have led to strong recommendations from major pediatric organizations (e.g., European Society for Pediatric Gastroenterology Hepatology and Nutrition, American Academy of Pediatrics) for the use of human milk as the primary nutritional source for preterm infants [14,15].

However, the nutritional needs of rapidly growing preterm infants often exceed what can be provided by human milk alone. Preterm infants, particularly those born before 32 weeks gestation or with birth weights less than 1500 g, require higher concentrations of protein, energy, and minerals than are typically found in mature human milk [16]. To address this nutritional gap, fortification of human milk with additional nutrients has become standard practice in neonatal intensive care units worldwide [17].

Traditionally, human milk fortification has relied on bovine milk-derived products that provide concentrated sources of protein, carbohydrates, fats, vitamins, and minerals. While these fortifiers effectively address the nutritional deficits of human milk for preterm infants, their use introduces foreign proteins and other components that may compromise some of the protective benefits of exclusive human milk feeding [18]. Studies have shown that even small amounts of bovine protein exposure can alter the gut microbiome, increase inflammatory markers, and potentially increase the risk of necrotizing enterocolitis compared to exclusive human milk feeding [19].

The development of human milk-derived fortifiers represents a significant advancement in neonatal nutrition, offering the potential to achieve adequate nutritional supplementation while maintaining the benefits of exclusive human milk feeding [20].

However, the production of human milk-derived fortifiers faces significant challenges related to supply, processing, storage, and cost [21]. Traditional liquid donor human milk requires frozen storage and has limited shelf life, creating logistical challenges for widespread implementation.

Freeze-drying, or lyophilization, offers a promising solution to many of these challenges. This preservation technology removes water from frozen milk through sublimation, creating a stable powder with extended shelf life that can be stored at ambient temperature [22]. The freeze-drying process preserves most nutritional and bioactive components while dramatically reducing storage and transportation costs [23,24]. When reconstituted with water, freeze-dried human milk closely resembles fresh milk in composition and functionality [25].

A recent comprehensive scoping review by Sproat et al. identified 48 studies examining the effects of freeze-drying on human milk composition and clinical outcomes [22]. This systematic analysis revealed that while freeze-drying affects certain components of human milk, including fat globule size and some bioactive factors, the overall nutritional profile remains largely intact. Importantly, the review highlighted that freeze-drying significantly decreases human milk fat globule size, potentially improving bioavailability, while preserving total protein content and human milk oligosaccharides [22]. However, the review also identified reductions in certain bioactive components, including immunoglobulin A, bile-stimulated lipase, and vitamin C content, though the clinical significance of these changes in the context of fortification remains to be fully elucidated.

In addition, clinical data on the safety, tolerability, and efficacy of freeze-dried human milk fortifiers in preterm infants remain limited, with the Sproat review emphasizing the critical need for more comprehensive clinical studies to establish safety and efficacy across different preterm populations [22].

The unique nutritional profile of freeze-dried human milk fortifiers presents both opportunities and challenges for clinical implementation. Unlike bovine protein-based fortifiers, which are specifically formulated to provide optimal protein-to-energy ratios for preterm infants, freeze-dried human milk maintains the natural composition of donor milk, including higher fat content and different macronutrient ratios. This composition may be particularly suitable for more mature preterm infants who can tolerate higher energy density feeds but may be less optimal for very low birth weight infants who require carefully balanced nutritional ratios. The scoping review noted that achieving recommended macronutrient intakes for preterm infants using freeze-dried human milk fortification may be challenging, with different lyophilization factors affecting macronutrient concentrations unequally [22].

As part of our quality improvement initiative to achieve exclusive human milk feeding for preterm infants, we introduced a freeze-dried high-temperature short-time pasteurized human milk fortifier into our routine clinical practice for preterm infants with gestational age ≥30 weeks. This preliminary study aims to evaluate the safety, tolerability, and growth outcomes of freeze-dried human milk fortification compared to conventional bovine protein-based fortification in a matched cohort of preterm infants.

## 2. Materials and Methods

### 2.1. Study Design and Setting

This prospective observational study with a retrospective matched comparison cohort was conducted at the neonatal intensive care unit of the University Children’s Hospital of Nuremberg, Germany. The study protocol was registered with the institutional research board (#SZ_D_083.20-IX-1) and conducted in accordance with the Declaration of Helsinki and Good Clinical Practice guidelines. The exposed cohort was recruited prospectively from September 2020 to August 2021, while the retrospective matched comparison cohort was selected from infants treated between September 2019 and September 2020. Written informed consent was obtained from parents or legal guardians of all participants in the exposed cohort.

### 2.2. Participants

#### 2.2.1. Inclusion Criteria

Preterm infants with gestational age ≥30 weeks who were fed with mother’s own milk and had an anticipated duration of mother’s own milk fortification of at least 14 days before discharge were eligible for inclusion. Infants were enrolled once enteral intake reached 70 mL/kg/d, indicating readiness for fortification. The minimum study period was 14 days to allow adequate assessment of feeding tolerance and growth outcomes.

#### 2.2.2. Exclusion Criteria

Infants with gastrointestinal malformations, major congenital anomalies, necrotizing enterocolitis (Bell stage ≥ 2), history of abdominal surgery, or Gram-negative sepsis were excluded from the study. These exclusion criteria were designed to eliminate conditions that could independently affect feeding tolerance or growth outcomes.

#### 2.2.3. Matching Criteria

The retrospective comparison cohort was selected using strict matching criteria to ensure comparability with the exposed cohort. Matching parameters included birth weight (±10%), gestational age (±5 days), and feeding with fortified mother’s own milk during the study period. This matching strategy was designed to minimize confounding variables and enhance the validity of between-group comparisons.

### 2.3. Feeding Routines

#### 2.3.1. Freeze-Dried Human Milk Fortifier (Exposed Cohort)

Infants in the exposed cohort received AMMEVA Fortifier AF S50 (AMMEVA GmbH, Werder (Havel), Germany), a freeze-dried donor human milk fortifier from human milk that did not undergo Holder pasteurization, but microfiltration and high-temperature short-time pasteurization (10 s at 73 °C). The fortifier was introduced using a graduated dosing schedule to optimize tolerance: 1.6 g/100 mL mother’s own milk on study day 1, 3.2 g/100 mL on study day 2, and 4.8 g/100 mL from study day 3 onwards. At the full dose of 4.8 g/100 mL, AMMEVA Fortifier AF S50 (nutritional fact sheet from 2020) provided an additional 0.5 g protein (true protein), 2.7 g carbohydrates, 1.3 g fat, and 25.5 kcal per 100 mL of mother’s own milk.

Infants receiving AMMEVA Fortifier AF S50 were supplemented with vitamins and minerals according to the volume of fortified mother’s own milk consumed. Multi-vitamin supplementation was provided at 1 drop per 25 mL of fortified mother’s own milk (Multini^®^, Rainfarn Gesundheit, München, Germany). Calcium-glycerophosphate supplementation was administered at 1 capsule per 50 mL of fortified mother’s own milk, with capsules prepared in the hospital pharmacy containing 0.8 mmol calcium and 0.5 mmol phosphate. It should be noted that the first 12 infants enrolled did not routinely receive calcium-glycerophosphate supplementation, as this protocol was refined during the early phase of implementation. Additionally, 1 mg of vitamin K was administered every Monday as per standard clinical practice [26].

Before discharge, AMMEVA Fortifier AF S50 was replaced with conventional fortifier (Aptamil™ FMS, Milupa Nutricia GmbH, Frankfurt, Germany) using a two-day transition period to ensure smooth transfer to post-discharge nutrition. After discharge, continued fortification with FMS was recommended until 50 weeks postmenstrual age.

#### 2.3.2. Bovine Protein-Based Fortifier (Matched Cohort)

Infants in the comparison cohort received Aptamil™ FMS, a standard bovine protein-based human milk fortifier. The fortifier was administered at half dose for one day, followed by the recommended full dose (4.4%) until 50 weeks postmenstrual age. The full dose added 1.1 g protein, 2.7 g carbohydrates, 0 g fat and 15 kcal per 100 mL of mother’s own milk. No additional supplementation beyond the fortifier was routinely provided to the comparison cohort.

#### 2.3.3. Feed Preparation and Administration

All feedings were prepared in a dedicated milk preparation unit located outside the neonatal intensive care unit, with access restricted to trained dieticians. This centralized approach ensured standardized preparation procedures and quality control. Each infant received their own mother’s milk. For practical handling, mother’s own milk was pooled for each mother individually to obtain batches with sufficient volumes for 24 h periods, and fortification was performed using a standard fortification approach [21].

Individual feedings were prepared from the fortified batches and stored under refrigeration until administration. Before feeding, milk bottles were warmed using a standardized bottle warmer, and the contents were gently mixed to ensure uniform distribution of the fortifier. This preparation protocol was designed to maintain the integrity of bioactive components while ensuring consistent nutritional delivery.

#### 2.3.4. Feeding Protocol

Enteral feeding was initiated according to standard clinical protocols, typically beginning on the first day of life at the discretion of the attending physician. Parenteral nutrition was provided as needed to supplement enteral intake during the advancement phase. Enteral feeding volumes were advanced by 20–30 mL/kg/d with a target intake of 160–170 mL/kg/d. Fortification was typically initiated when enteral feeding volumes reached 70–100 mL/kg/d, indicating adequate gastrointestinal tolerance and readiness for concentrated nutrition.

### 2.4. Outcome Measures

#### 2.4.1. Primary Outcomes

The primary outcomes included anthropometric measurements, feeding tolerance and safety parameters. Weight was measured daily, while length and head circumference were assessed weekly.

Feeding tolerance was assessed through systematic monitoring of gastric residuals, vomiting, spitting, abdominal distension, and abdominal tenderness. An abdominal score was developed specifically for this study, calculated from the sum of scores for abdominal distension (0 = not distended, 1 = moderately distended with visible bowel loops, 2 = severely distended with shiny abdominal skin and prolonged capillary refill time) and abdominal tenderness (0 = soft, 1 = slightly tense, 2 = tense, 3 = hard). An abdominal score of 0–1 was defined as normal/not bloated, 2 as moderately bloated and ≥3 was defined as abnormal.

Safety parameters included biweekly assessments of blood glucose and urea. Urinary calcium and phosphate levels were monitored weekly as per clinical routine. In the exposed cohort, also triglycerides were measured, and blood glucose was additionally monitored during fortifier introduction (days 1, 2, and 3).

#### 2.4.2. Secondary Outcomes

Secondary outcomes included body composition analysis, and nutritional intake assessment. Body composition (fat mass and fat-free mass) was measured in infants without respiratory support using the PEA POD^®^ Infant Body Composition System (COSMED USA Inc., Concord, CA, USA) [27,28]. Body composition measurement has been introduced in January 2021 in the University Children’s Hospital of Nuremberg, after the study had already begun. Therefore, body composition data is not available for the comparison cohort. Nevertheless, 25 of the 32 infants in the exposed cohort were able to undergo body composition analysis.

Nutritional intake was calculated daily in the exposed cohort, including feeding volumes, types of feeding, and intake of protein, carbohydrates, and fat. The intake of each macronutrient could be calculated in the exposed cohort due to daily milk analyses. Human milk composition was analyzed using the MIRIS mid-infrared milk analyzer (Miris, Uppsala, Sweden) on samples obtained from 24 h batches. Regular analysis of human milk composition had not been part of the routine care for infants born before September 2020. Therefore, in the comparison cohort, only total fluid intake had been measured consistently.

### 2.5. Statistical Analysis

Participant characteristics and outcomes are presented using descriptive statistics. Normally distributed parameters are reported as mean ± standard deviation, while non-normally distributed parameters are presented as median (1st quartile, 3rd quartile). Between-group comparisons for anthropometric and feeding volume outcomes were performed using Student’s *t*-test for normally distributed data or Mann–Whitney U test for non-normally distributed data. Categorical variables, including abdominal scores and feeding tolerance parameters, were compared using the Chi-square test.

Birth weight percentiles were calculated using established birth weight charts [29]. Statistical significance was set at *p* < 0.05 for all analyses. Data management was performed using a REDCap database [30], and statistical analyses were conducted using Microsoft Excel^®^ Office 365 (Redmond, WA, USA) and R software package version 4.2.0 (Vienna, Austria) [31].

## 3. Results

### 3.1. Participant Characteristics

A total of 64 preterm infants were enrolled in this study, with 32 infants in each cohort. The exposed cohort and the comparison cohort were well-matched for key demographic and clinical characteristics including mean gestational age and birth weights (Table 1). Birth length and head circumference were also not significantly different between cohorts.

Maternal characteristics showed some differences between cohorts. The comparison cohort had a higher prevalence of gestational diabetes (6 cases vs. 1 case), with five cases managed by diet and one requiring insulin therapy (Table 2). Conversely, the exposed cohort had higher rates of preeclampsia (4 cases vs. 2 cases) and HELLP syndrome (2 cases vs. 0 cases). However, these differences were not statistically significant. One case of maternal type 1 diabetes was present in the exposed cohort. No cases of chorioamnionitis were reported in either cohort.

No significant differences were observed in neonatal morbidities during the hospital stay between the two cohorts (Table 2). Importantly, no cases of necrotizing enterocolitis or other significant abdominal pathology were reported in either cohort. Early-onset sepsis was treated in two infants in the exposed cohort and three infants in the comparison cohort, representing a non-significant difference.

Respiratory support requirements were not different between groups. In the exposed cohort, 25 infants (78%) required oxygen therapy and respiratory support (nasal continuous positive airway pressure or high-flow nasal cannula) for a median duration of 7 (3, 13) days. In the comparison cohort, 27 infants (84%) required respiratory support for a median duration of 7 (4, 10) days.

### 3.2. Growth and Anthropometric Outcomes

**Figure 1:**
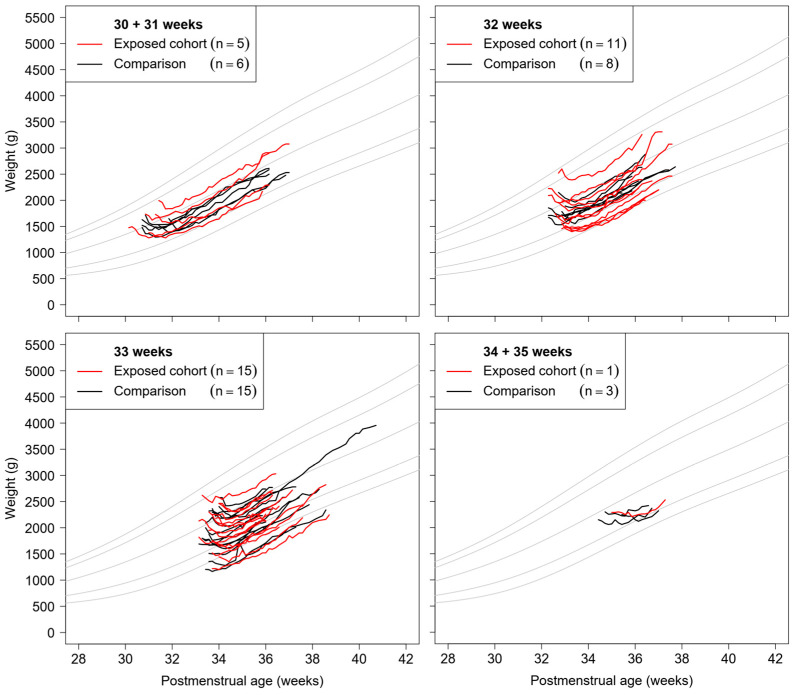
Weight trajectories of infants stratified by gestational age. Gray lines, reference centiles (3rd, 10th, 50th, 90th and 97th) according to Fenton et al. [29].

Growth trajectories in both cohorts followed patterns parallel to reference charts after the initial postnatal weight loss period, stratified by gestational age (Figure 1) [29]. Infants in the exposed cohort were discharged after 27 ± 7 days with a postmenstrual age of 36.5 ± 0.9 weeks and discharge weight of 2500 ± 380 g (Table 1). The comparison cohort had not significantly different outcomes with length of stay of 27 ± 10 days, postmenstrual age at discharge of 36.6 ± 1.2 weeks, and discharge weight of 2490 ± 360 g.

Body composition analysis was performed in 25 infants in the exposed cohort at 35.7 weeks postmenstrual age on average using air displacement plethysmography (Table 1). Fat mass averaged 290 ± 110 g, representing 12 ± 3% of total body weight, while fat-free mass was 2050 ± 240 g.

### 3.3. Feeding Tolerance

Feeding tolerance was not different between cohorts, based on the analysis of more than 3100 feedings fortified with freeze-dried donor milk and more than 2700 feedings fortified with a bovine protein-based fortifier. No significant differences were observed in feeding tolerance parameters or abdominal scores between cohorts (Table 3).

### 3.4. Safety-Related Blood Parameters and Urinary Electrolyte Parameters

Safety monitoring during the introduction phase revealed stable blood glucose levels throughout the graduated dosing period. Mean blood glucose was 82 mg/dL on days 1 and 2 in the exposed cohort, and 83 mg/dL on day 3 when full fortification was achieved. During the maintenance phase, mean blood glucose remained stable at a mean of 86 mg/dL in the exposed cohort, not significantly different to the comparison cohort (Table 4).

Triglyceride levels in the exposed cohort averaged 105 ± 36 mg/dL. Mean urea levels in the exposed cohort and in the comparison cohort were not significantly different.

Urinary calcium and phosphate monitoring revealed patterns related to supplementation strategies. Of the 32 infants in the exposed cohort, 20 received additional calcium-phosphate supplementation while 12 did not receive supplementation. Overall urinary calcium in the exposed cohort was 3.3 ± 4.6 mmol/L, with higher levels in supplemented infants compared to unsupplemented infants (Table 4).

Urinary phosphate levels showed more pronounced differences. In the exposed cohort, supplemented infants showed higher levels compared to unsupplemented infants, though not statistically significant. The comparison cohort demonstrated significantly higher urinary phosphate levels compared to unsupplemented infants of the exposed cohort.

### 3.5. Nutritional Intake and Feeding Advancement

The time to achieve full enteral feeding was not significantly different between cohorts (Table 5). Fortification was initiated at postnatal ages that were not significantly different in both cohorts.

The exposed cohort had lower total fluid intake compared to the comparison cohort (Table 5).

## 4. Discussion

Our observational study suggests that the fortification of human milk with a fortifier made from lyophilized high-temperature short-time pasteurized donor milk may be feasible, well-tolerated, and allows for adequate growth in a population of relatively mature preterm infants. To our knowledge, this is the first study to evaluate the use of a lyophilized human milk-based fortifier produced with high-temperature short-time pasteurization rather than the more commonly used Holder or low-temperature long-time pasteurization, which have been applied in previous studies using either liquid or freeze-dried human milk-derived fortifiers. Our preliminary findings indicate that donor milk fortification could represent a promising, entirely human milk-based nutritional strategy and a potential alternative to conventional bovine milk-based human milk fortifiers, but confirmation in larger, multicenter trials is required.

In the present study, the two fortification strategies did not differ from each other regarding clinical outcomes, as shown by the absence of significant differences in anthropometric measures, neonatal morbidities, and discharge parameters. The exposed and comparison cohorts were well-matched at baseline. This ensured comparable baseline growth potential between the two cohorts. The lack of difference in respiratory support requirements confirmed comparable clinical acuity between cohorts. At discharge, no significant differences in weight, length, and head circumference were observed, suggesting that both fortification strategies supported adequate growth and development during the critical preterm period. Importantly, body composition analysis in the exposed cohort indicated appropriate growth quality and normal body composition development [32,33,34]. Feeding tolerance was not compromised in the exposed cohort, despite the higher energy density of the feeds fortified with freeze-dried donor milk, suggesting good gastrointestinal tolerance. Biochemical markers further demonstrated safety: triglyceride levels remained within normal ranges despite the higher fat content of the freeze-dried human milk fortifier [35], and mean urea levels indicated appropriate protein metabolism in both cohorts [36]. Differences in urinary phosphate levels, with significantly higher values in the comparison cohort compared to unsupplemented infants of the exposed cohort, may reflect fortifier-related differences in phosphate handling. However, the wide variability in urinary electrolyte levels observed in both cohorts is consistent with the known heterogeneity in mineral metabolism among preterm infants and the influence of multiple factors including gestational age, postnatal age, and individual variation in renal function. Finally, the lower fluid intake observed in the exposed cohort may reflect the higher energy density of the feeds fortified with freeze-dried human milk, leading to earlier satiety and reduced volume tolerance.

The key advantage of a donor milk fortifier lies in its exclusive use of human milk. This avoids the intake of proteins from cow’s milk, which can potentially reduce the risk of feeding intolerance and necrotizing enterocolitis [37], one of the most feared complications in preterm infants. In our study, we did not observe differences in feeding intolerance between the cohorts, nor in neonatal morbidities such as necrotizing enterocolitis, early-onset sepsis, or respiratory support requirements. However, the sample size of this study was limited. Furthermore, lyophilization (freeze-drying) and high-temperature short-time pasteurization are gentler preservation methods than Holder pasteurization. Compared to Holder pasteurization, which is the standard for processing donor milk, freeze-drying better preserves important bioactive components such as immunoglobulins, lactoferrin, and lysozyme [22,38]. Studies by Martysiak-Żurowska et al. and Cortez & Soria demonstrate that the concentration of nutrients and antioxidants in lyophilized human milk remains largely stable, maximizing the nutritional value for the delicate preterm infant [39,40].

However, the use of human milk-based fortifiers is not without limitations. These products are very costly, require large amounts of donor milk for production, and remain supported by limited efficacy data [21].

A central point of our observations and a significant limitation of the donor milk fortifier used here is the protein-to-energy ratio. The fortifier we used was produced by freeze-drying mature donor milk and therefore has the natural protein-to-energy ratio of human milk. This ratio appears to be sufficient to ensure adequate growth for the cohort of more mature preterm infants we studied (i.e., after 30 weeks of gestation) [41], as previous data also showed [42].

Fortifying with a lyophilized donor-milk fortifier has a role in more mature preterm and term infants who have higher energy requirements or need fluid restriction. For very immature preterm infants (very or extremely low birth weight infants), however, this ratio represents a significant limitation. This most vulnerable patient group has a significantly higher protein requirement per calorie (i.e., >3.1 g/100 kcal) for optimal catch-up growth and organ development than what mature human milk (approx. 1.9 g/100 kcal) or a simple lyophilized donor milk fortifier (fortifier in this study: 1.9 g/100 kcal) can provide [43,44,45,46]. Current guidelines of the European Society for Paediatric Gastroenterology, Hepatology and Nutrition recommend a protein intake of up to 4.5 g/kg/d [14]. To achieve this target value solely by increasing the amount of a simple lyophilized donor milk fortifier, an excessive amount of energy, fat, and carbohydrates would have to be administered. This would not only increase the risk of metabolic problems (hyperglycemia, acidosis) and undesirable fat deposition but also exceed the volume tolerance of the immature gastrointestinal tract.

This leads to the compelling need to develop a novel human milk fortifier tailored to the needs of the smallest preterm infants. Instead of merely freeze-drying donor milk, a product must be created for this patient group in which protein is specifically enriched. The amount of fat and carbohydrates need to be reduced. Technologically, this could be achieved by separating and concentrating protein fractions from a pool of donor milk before the lyophilization process. Only such a protein-enriched human milk fortifier could meet the metabolic demands of the most immature preterm infants without burdening them with excess energy and fluid [14,41].

Our study has limitations, including its observational design and small sample size. In addition, although the potential impact of different fortifiers on the gut microbiome is an important consideration, this aspect was not investigated in the present study. Another major limitation is the use of retrospective controls. This study originated from a change in clinical routine as part of a quality improvement initiative. Consequently, infants fed according to the former feeding protocol could only be included retrospectively, as no patients received the old protocol after September 2020. A randomized controlled trial was not feasible in this context, and no concurrent control group was available. These factors further emphasize the preliminary nature of our work. Nevertheless, it provides important proof-of-concept for lyophilized high-temperature short-time pasteurized donor milk fortification in a specific subgroup of preterm infants.

## 5. Conclusions

This study demonstrates that freeze-dried high-temperature short-time pasteurized human milk fortification is safe and well-tolerated in preterm infants ≥30 weeks gestational age and achieves anthropometric outcomes not significantly different to conventional bovine protein-based fortification. However, the suboptimal protein-to-energy ratio inherent in freeze-dried human milk, which reflects the natural composition designed for term infant nutrition, presents challenges for optimal preterm infant nutrition. The development of human milk-derived fortifiers with higher protein content and lower energy density would better serve the needs of very and extremely low birth weight infants, representing an important direction for future product development and clinical research in neonatal nutrition.

## Figures and Tables

**Table 1:** Anthropometric Parameters at Birth and Discharge and Body Composition.

Parameter	Exposed Cohort	Comparison Cohort	*p*-Value
Birth parameters	*n* = 32	*n* = 32	
C-section/spontaneous birth	20/12	19/13	1.0
Sex (male)	20	18	0.8
Gestational age (weeks)	32.8 ± 1.0	33.0 ± 1.2	0.53
Weight (g)	1900 ± 380	1840 ± 370	0.52
Weight percentile	48 ± 28	44 ± 23	0.54
Length (cm)	43.3 ± 2.6	43.0 ± 3.0	0.68
Head circumference (cm)	30.5 ± 1.5	30.6 ± 1.3	0.78
Discharge parameters	*n* = 32	*n* = 32	
Length of stay (days)	27 ± 7	27 ± 10	0.95
Postmenstrual age (weeks)	36.5 ± 0.9	36.6 ± 1.2	0.73
Weight (g)	2500 ± 380	2490 ± 360	0.91
Weight percentile	30 ± 24	26 ± 18	0.45
Length (cm)	46.5 ± 2.5	47.1 ± 2.3	0.34
Head circumference (cm)	32.5 ± 1.4	32.5 ± 1.5	0.98
Body composition	*n* = 25		
Postmenstrual age (weeks)	35.7		
Body weight (g)	2310 ± 420		
Length (cm)	45.4 ± 2.3		
Head circumference (cm)	31.8 ± 1.2		
% Fat mass	12 ± 3		
Fat mass (g)	290 ± 110		
Fat-free mass (g)	2050 ± 240		

Values are presented as mean ± standard deviation, except for mode of delivery (C-section/spontaneous birth) and sex (male), which are presented as absolute numbers. Postmenstrual age at the time of body composition measurement is presented as a mean value only.

**Table 2:** Pregnancy Risks and Neonatal Morbidities.

Parameter	Exposed Cohort (*n* = 32)	Comparison Cohort (*n* = 32)	*p*-Value
Pregnancy risks			
Gestational diabetes (diet-controlled/insulin)	1 (0/1)	6 (5/1)	0.1
Maternal diabetes Type 1	1	0	1.0
Preeclampsia	4	2	0.67
HELLP syndrome	2	0	0.49
Chorioamnionitis	0	0	n.a.
Neonatal morbidities			
Necrotizing enterocolitis	0	0	n.a.
Early-onset sepsis	2	3	1.0
Oxygen therapy and respiratory support	25 (78%)	27 (84%)	0.75
Duration of respiratory support (median, 1st quartile, 3rd quartile)	7 (3, 13)	7 (4, 10)	1.0

Values are presented as absolute numbers, unless otherwise stated. HELLP, hemolysis, elevated liver enzymes, and low platelet count; n.a., not applicable.

**Table 3:** Feeding Intolerance and Abdominal Score.

Parameter	Exposed Cohort (*n* = 32)	Comparison Cohort (*n* = 32)	*p*-Value
Feeding intolerance			
Gastric residual (%)	0 (0, 0)	0 (0, 0)	n.a.
Spitting (%)	3 (0, 8)	2 (0, 6)	0.55
Emesis (%)	0 (0, 0)	0 (0, 0)	n.a.
Abdominal score			
Days with normal score (%)	94 (85, 100)	100 (94, 100)	0.98
Days with moderate bloating (%)	5 (0, 13)	0 (0, 6)	0.96
Days with abnormal score (%)	0 (0, 3)	0 (0, 3)	n.a.

Values are presented as median (1st quartile, 3rd quartile). n.a., not applicable.

**Table 4:** Safety-Related Blood Parameters and Urinary Calcium and Phosphate Levels.

Parameter	Exposed Cohort			Comparison Cohort	*p*-Value
	*n* = 32	Without CaPh-suppl. *n* = 12	with CaPh-suppl. *n* = 20	*n* = 32	
Blood parameters					
Glucose (mg/dL)	86 ± 19			87 ± 17	0.17
Triglycerides (mg/dL)	105 ± 36				
Urea (mg/dL)	21 ± 12			22 ± 12	0.62
Urinary levels					
Calcium (mmol/L)	3.3 ± 4.6	2.6 ± 2.2	3.8 ± 5.7	2.5 ± 1.8	0.64
Phosphate (mmol/L)	3.8 ± 4.6	2.3 ± 2.1	4.7 ± 5.4	7.8 ± 5.4	1.0

Values are presented as mean ± standard deviation. CaPh-suppl., calcium-glycerophosphate supplementation.

**Table 5:** Early Feeding Advancement, Mother’s Own Milk Composition and Nutritional Intake in the Exposed Cohort.

Parameter	Exposed Cohort (*n* = 32)	Comparison Cohort (*n* = 32)	*p*-Value
Early feeding advancement			
Days until full enteral feeding	5.0 ± 1.3	5.2 ± 1.5	0.43
Start of fortification (day of life)	6.8 ± 1.8	6.8 ± 2.7	1.0
Mother’s own milk composition			
Energy (kcal/100 mL)	67.7 ± 9.1		
Protein (g/100 mL)	1.5 ± 0.3		
Carbohydrates (g/100 mL)	6.8 ± 0.4		
Fat (g/100 mL)	3.8 ± 1.0		
Protein per energy (g/100 kcal)	2.3 ± 0.3		
Nutritional intake			
Energy intake (kcal/kg/d)	141 ± 18		
Fluid intake (mL/kg/d)	151 ± 12	158 ± 10	0.04
Protein (g/kg/d)	3.1 ± 0.5		
Carbohydrates (g/kg/d)	14.4 ± 1.2		
Fat (g/kg/d)	7.8 ± 1.7		
Protein per energy (g/100 kcal)	2.2 ± 0.2		

Values are presented as mean ± standard deviation.

## Data Availability

Raw data are unavailable due to ethical restrictions.

## Abbreviations

The following abbreviations are used in this manuscript:

CaPh-suppl.	calcium–glycerophosphate supplementation
HELLP	hemolysis, elevated liver enzymes, and low platelet count
